# Acid-Induced Epimerization of Eudesmin to Epieudesmin: Rapid Bactericidal and Antibiofilm Activity Against *Helicobacter pylori*

**DOI:** 10.3390/ijms27146295

**Published:** 2026-07-15

**Authors:** Cristian Parra-Sepúlveda, Kimberly Sánchez-Alonzo, José Becerra, Leandro Ortiz, Viviana Burgos, Cecilia Villegas, Felipe A. Sanhueza, Benjamín Oporto, Cristobal Goic, Apolinaria García-Cancino, Cristian Paz

**Affiliations:** 1 Laboratory of Bacterial Pathogenicity, Faculty of Biological Sciences, Universidad de Concepción, Concepcion 4030000, Chile; cparras@udec.cl (C.P.-S.); kimsanchez@udec.cl (K.S.-A.); cmanzor2018@udec.cl (C.G.); 2 Doctorado en Ciencias Mención Biología Celular y Molecular Aplicada, Universidad de La Frontera, Temuco 4811230, Chile; 3 Departamento de Botánica, Facultad de Ciencias Naturales y Oceanográficas, Universidad de Concepcion, Concepcion 4070386, Chile; jbecerra@udec.cl; 4 Instituto de Ciencias Químicas, Facultad de Ciencias, Universidad Austral de Chile, Casilla 567, Valdivia 5090000, Chile; leandro.ortiz@uach.cl; 5 Escuela de Tecnología Médica, Centro de Investigación en Prevención y Cuidados de la Salud (+SALUD), Facultad de Salud, Universidad Santo Tomás, Temuco 4780000, Chile; vburgos7@santotomas.cl; 6 Departamento de Ciencias Químicas y Biológicas, Facultad de Recursos Naturales, Universidad Católica de Temuco, Rudecindo Ortega, Temuco 4781509, Chile; cecilia.villegas@uct.cl; 7 Laboratory of Natural Products & Drug Discovery, Department of Basic Sciences, Faculty of Medicine, Universidad de La Frontera, Temuco 4811230, Chile; felipesanhueza2017@gmail.com (F.A.S.); b.oporto02@ufromail.cl (B.O.)

**Keywords:** lignans, eudesmin, epieudesmin, *Helicobacter pylori*, biofilm, epimerization, antibacterial resistance, furanofuran lignan

## Abstract

*Helicobacter pylori* infection is associated with severe gastroduodenal pathologies. This study evaluated the bactericidal and antibiofilm activities of eudesmin, a furanofuran lignan from *Araucaria araucana*, and its acid-induced epimer, epieudesmin, against clinical isolates and the *H. pylori* ATCC 43504 strain. Virulence profiles and antibiotic susceptibility (CLSI/EUCAST) were determined. Antibacterial effects were assessed via minimum inhibitory concentration (MIC), checkerboard assays, time-kill kinetics, and urease activity. Biofilm reduction and morphological alterations were analyzed using crystal violet staining and scanning electron microscopy, respectively. Cytotoxicity was evaluated in HepG2 cells. Clinical strains exhibited distinct virulence and multidrug-resistance patterns. Both lignans displayed rapid antibacterial activity (MIC: 0.78–3.12 µg/mL) without antagonistic interactions with co-administered antibiotics and significantly reduced biofilm biomass (*p* < 0.0001). Eudesmin and epieudesmin effectively inhibit *H. pylori* growth and biofilm formation, while sublethal concentrations disrupted biofilm integrity and induced a bacillary-to-coccoid transition. Toxicity of both compounds was evaluated in HepG2 cells till 50 µg/mL showing a similar pattern, reducing cell viability up to 30% at almost 25 µg/mL, highlighting its potential as a non-antibiotic complementary therapeutic agent, although further in vivo and clinical studies are warranted to confirm its therapeutic applicability.

## 1. Introduction

*Helicobacter pylori* is a Gram-negative, microaerophilic bacterium that colonizes the human gastric epithelium [1]. This infection is associated with the development of several diseases such as atrophic gastritis, peptic ulcers, gastric mucosa-associated lymphoid tissue (MALT) lymphoma, and gastric adenocarcinoma [2,3,4]. It is estimated that *H. pylori* infection affects approximately 50% of the world’s population [5]. However, its prevalence varies according to geographical areas, being higher in less developed countries [6]. Therefore, effective therapy is needed to reduce its global incidence. For decades, the standard first-line treatment for this infection has been triple therapy, which includes a proton pump inhibitor (PPI), clarithromycin, and either amoxicillin or metronidazole [7]. Although this therapy has been useful in reducing pathologies associated with *H. pylori*, its high cost, patient non-adherence to therapy, and the emergence of resistant strains have contributed to a decrease in the effectiveness of treatment [8,9,10].

Notably, due to its well-established association with gastric cancer, *H. pylori* was classified as a Group 1 human carcinogen by the International Agency for Research on Cancer (IARC) in 1994 [11]. *H. pylori* produces a range of enzymes, including oxidase, catalase, urease, and carbonic anhydrase [12,13], which help neutralize the acidic gastric environment, enabling the bacterium to survive in the gastric epithelium for decades [14]. In particular, urease has been shown to neutralize gastric HCl [12].

After colonizing the gastric mucosa, *H. pylori* secrete proteins, polysaccharides, extracellular DNA (eDNA), and other molecules to create extracellular polymeric substances (EPSs) forming biofilms [15]. Biofilm-forming colonies are highly resistant to external environmental stressors, including exposure to antibiotics [16]. It is well established that when bacteria develop biofilms, their resistance to antibiotics increases by 10- to 1000-fold [17]. *H. pylori* biofilm formation is probably the main cause of long-term chronic infection, multidrug resistance, and treatment failure [18]. Therefore, it is necessary to develop new alternatives that could increase treatment effectiveness, for example by using natural products [19,20,21].

The World Health Organization (WHO) has defined herbal medicine as a plant-derived material containing raw or processed ingredients from one or more plants that provide therapeutic health benefits [22]. Advances in science and modern technology have greatly contributed to the development of the phytopharmaceutical industry, allowing the use of plant derivatives for the development of new medicines against communicable and non-communicable diseases in humans [23]. Lignans are a group of non-flavonoid diphenolic compounds that act as antioxidants and have shown antimicrobial activity against pathogenic bacteria and fungi [19,23,24]. Among them, eudesmin is a natural furofuran lignan found in fruits and seeds of various plants of Apiaceae, Magnoliaceae, Ochnaceae, Acanthaceae, Rutaceae, Araucariaceae, and Leguminosae families [25]. Eudesmin can induce neurite growth from PC12 cell neurons by stimulating upstream signaling of mitogen-activated protein kinase, protein kinase C, and protein kinase A pathways [23].

Eudesmin significantly inhibited TNF-α production by lipopolysaccharide (LPS)-stimulated murine macrophages RAW264.7 without showing cytotoxicity, suggesting that eudesmin can inhibit TNF-α without interfering with normal cell function, in a dose-dependent manner [26]. Moreover, lignans isolated from *Magnolia fargesii* flower buds, including eudesmin, also inhibited TNF-α production with an IC_50_ = 51 μM [27]. Given that the pathophysiological consequences of various diseases result from excessive TNF-α release and T-cell proliferation, it is plausible that eudesmin could have beneficial effects in clinical practice [26]. The biological potential of eudesmin in drug transport by P-glycoprotein has been investigated [28]. Yang et al. (2018) investigated the effects of eudesmin, extracted from *Fatsia polycarpa*, on epithelial damage caused by *H. pylori*, as well as *H. pylori* colonization in vitro in human gastric adenocarcinoma (AGS) cells and in vivo in C57BL/6 mice [29]. Therefore, the available evidence indicates that eudesmin might have therapeutic potential against *H. pylori*, supporting its possible use as an adjunct agent in the management of gastric infections. Previous studies investigated the bromination of eudesmin in acid media looking for anti-Candida agents [30]. The aim of this study was to (i) evaluate the stability of eudesmin under gastric-like pH conditions and characterize the resulting epimer, epieudesmin; and (ii) assess the bactericidal and antibiofilm activities of both compounds against *H. pylori* clinical isolates, including multidrug-resistant strains, as potential complementary agents to conventional antibiotic therapy.

## 2. Results

### 2.1. Epimerization of Eudesmin

**Scheme 1:**
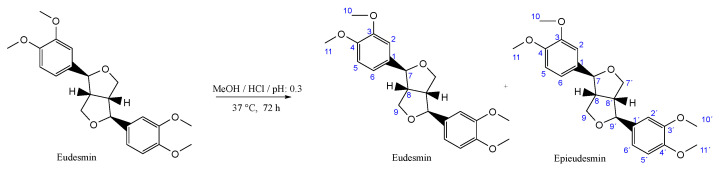
Acid-catalyzed epimerization of eudesmin in aqueous solution yields a ~1:1 mixture of eudesmin and epieudesmin.

Eudesmin is a symmetrical furofuran lignan that, under acidic conditions, undergoes epimerization via protonation of the furan ring, yielding a mixture of diastereomers Scheme 1. The epimer epieudesmin was isolated by column chromatography with purity exceeding 98%.

**Figure 1:**
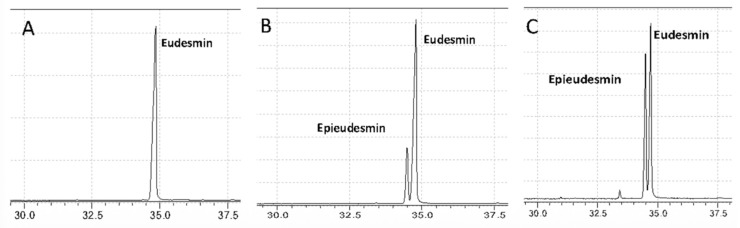
GC–MS chromatograms of the epimerization of eudesmin over time. (**A**) After 24 h at pH 3, 2, or 1, a single peak corresponding to eudesmin was observed (RT = 34.914 min). (**B**) At pH 0.3 after 24 h, the formation of epieudesmin was detected (RT = 34.510 min). (**C**) After 72 h at pH 0.3, the reaction approached equilibrium, showing two peaks corresponding to eudesmin (RT = 34.907 min) and epieudesmin (RT = 34.493 min).

The epimerization of eudesmin was evaluated by GC–MS under four different pH conditions (pH 3, 2, 1, and 0.3). At pH 3, 2, and 1, no detectable conversion was observed, and the chromatograms showed only the starting material, eudesmin (RT = 34.914 min; Figure 1A). In contrast, when the reaction was conducted at pH 0.3 and 37 °C for 24 h, the formation of epieudesmin was detected, with a retention time of 34.510 min (Figure 1B). Prolonged reaction times led to a progressive approach to equilibrium between both epimers, which was nearly achieved after 72 h (Figure 1C).

### 2.2. Determination of Virulence Genes

The molecular characterization of virulence gene profile of *H. pylori* strains was evaluated by PCR, and it is resumed in Table 1.

### 2.3. Antibacterial Activity

The bactericidal activity of eudesmin and epieudesmin was evaluated against four strains of *H. pylori*, including the ATCC 43504 and three clinical strains isolated from Chilean patients. The results are summarized in Table 2.

The reference strain ATCC 43504 was susceptible to all antibiotics tested except metronidazole (MIC = 64 µg/mL), while clinical strain 72A was susceptible to all five antibiotics. In contrast, strain 216A exhibited a multidrug-resistant (MDR) phenotype, showing resistance to clarithromycin, metronidazole, and levofloxacin. Similarly, strain 239A was resistant to clarithromycin and levofloxacin. Although all bacterial strains remained susceptible to amoxicillin and tetracycline, the presence of these diverse resistance profiles in clinical isolates underscores the difficulty of treating *H. pylori* infections and warrants the evaluation of new therapeutic alternatives (Table 3).

To evaluate the potential application of eudesmin and epieudesmin, we observed their effects when combined with the common antibiotics used in *H. pylori* therapies and in areas where antibiotic resistance is observed (checkerboard pattern). The results showed that both molecules had no effect when combined with CLR, MTZ, and LEV, indicating that these molecules do not negatively interfere with antibiotic activity, thus supporting their potential use in combination with antibiotics (Table 3).

**Figure 2:**
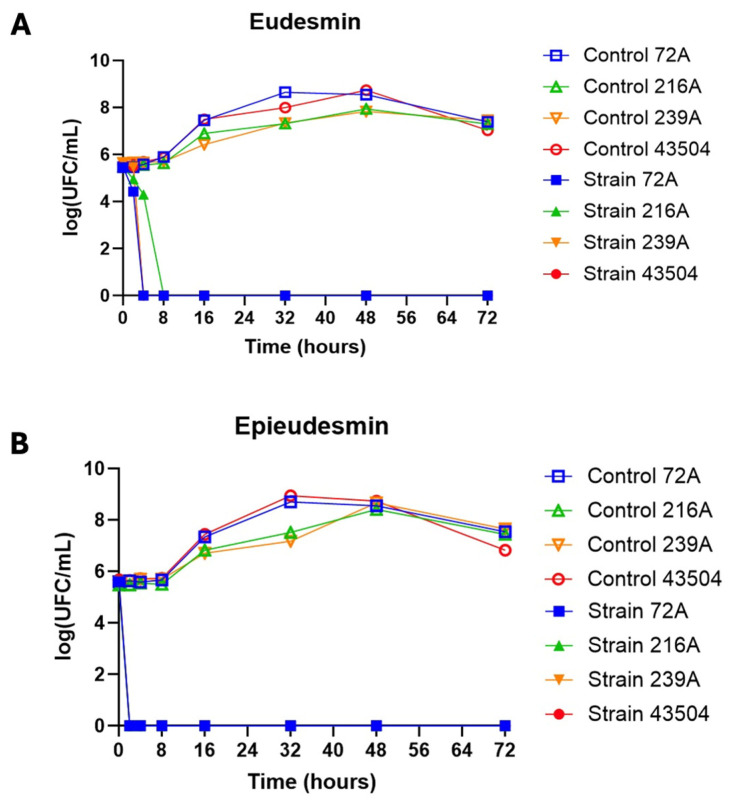
Bacterial counts of *H. pylori* strains 72A, 216A, 239A, and ATCC 43504 incubated at 37 °C under microaerophilic conditions using a McFarland 2 inoculum (*n* = 3). (**A**) Viable *H. pylori* count following exposure to eudesmin at different time points. (**B**) Viable *H. pylori* count following exposure to epieudesmin at different time points.

The antibacterial activity of eudesmin and epieudesmin against *H. pylori* was assessed by death kinetics assays (Figure 2). Both compounds were tested at half of their minimum inhibitory concentration (MIC) in Brain Heart Infusion (BHI) medium, and viable bacteria were quantified by microdrop counting as described in the Methods section. For *H. pylori* strains 72A, 239A, and ATCC 43504 treated with eudesmin, bacterial growth was detected up to 2 h after assay initiation. For Eudesmin (Figure 2A), marked bactericidal activity was observed. At 4 h of exposure, CFU counts fell below the limit of detection (LOD < 10 CFU/mL) in most strains. Clinical strain 216A showed transient bacterial persistence, reaching undetectable levels at 6 h. The robustness of this elimination kinetics was confirmed by extended monitoring at 8, 16, 32, 48, and 72 h; at all intervals, counts remained below the LOD, confirming the absence of bacterial growth.

In contrast, treatment with epieudesmin (Figure 2B) exhibited significantly faster elimination kinetics. After the initial inoculum (t = 0), no viable bacteria were detected in any of the four strains evaluated after 2 h of treatment. This state of total elimination remained constant throughout the observation period up to 72 h, corroborating an irreversible and early bactericidal effect with no evidence of cellular recovery under the tested conditions.

### 2.4. Biofilm Inhibition

**Figure 3:**
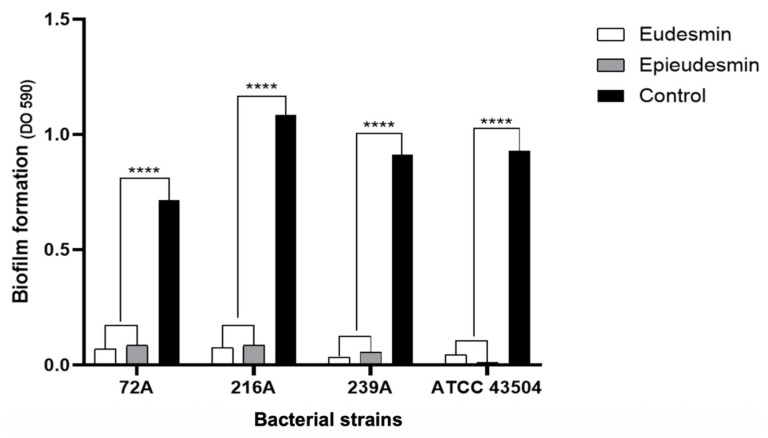
*H. pylori* biofilm formation by strains 72A, 216A, 239A, and ATCC 43504, quantified as total biomass by Crystal Violet staining. The *Y*-axis represents the Optical Density measured at 590 nm (${\mathrm{OD}}_{590}$) on glass surfaces inoculated with a McFarland 2 suspension; higher values indicate a greater accumulation of biofilm biomass. White bars represent biofilm formation in the presence of eudesmin at half of the minimum inhibitory concentration (1/2 MIC), gray bars represent biofilm formation in the presence of epieudesmin at 1/2 MIC, and black bars correspond to untreated controls. Data are expressed as mean ± SD from three independent experiments performed in duplicate. **** indicates statistically significant differences (p<0.0001), as determined by one-way ANOVA followed by Bonferroni’s post hoc test.

Biofilm biomass reduction was evaluated using half of the minimum inhibitory concentration (½ MIC) of eudesmin or epieudesmin for each *H. pylori* strain and compared with untreated controls. The results demonstrated that both compounds significantly inhibited biofilm biomass reduction in all bacterial strains tested (*p* < 0.0001), as shown in Figure 3.

**Figure 4:**
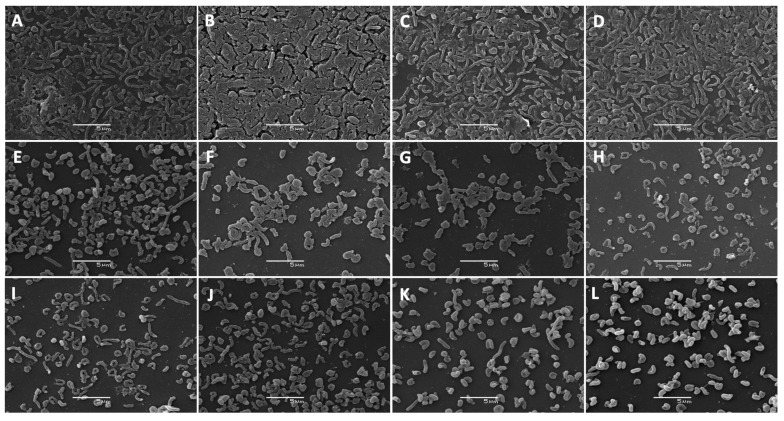
Scanning electron microscopy (SEM) images of *H. pylori* biofilm formation. Representative micrographs of untreated control biofilms are shown in panels (**A**–**D**). The effects of eudesmin on *H. pylori* biofilm architecture are shown in panels (**E**–**H**), while the effects of epieudesmin are shown in panels (**I**–**L**). Biofilms were formed using an initial inoculum of 1.5 × 10^8^ CFU/mL and visualized after 24 h of incubation. Scale bar: 5 μm.

In addition to the quantification of biofilm by crystal violet (Figure 3), the morphology of *H. pylori* biofilm was evaluated by scanning electron microscopy (SEM) following treatment with eudesmin and epieudesmin at ½ MIC (Figure 4). The results of this study demonstrated that both compounds modified the morphology of *H. pylori* from bacillary to coccoid, along with a loss of biofilm integrity. This supports the previous results of biofilm biomass reduction quantified with CV.

### 2.5. Urease Activity Assay

To explore the potential mechanism of action of eudesmin and epieudesmin, their effects on *H. pylori* urease activity were evaluated using half of the minimum inhibitory concentration (½ MIC) for each bacterial strain. The results showed that neither eudesmin nor epieudesmin inhibited urease activity, indicating that these compounds do not interfere with urease function under the experimental conditions employed.

### 2.6. Toxicity

**Figure 5:**
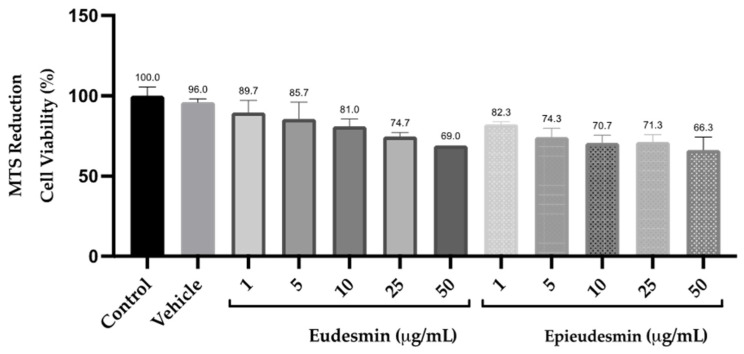
Effect of eudesmin and epieudesmin on HepG2 cell viability until 50 µg/mL. Cell viability was assessed by MTS assay compared to Control (untreated cells) and control vehicle: DMSO 0.1%. The data are expressed as mean ± standard deviation, *n* = 3.

The cytotoxic effects of eudesmin and epieudesmin were evaluated in HepG2 hepatic cells using the MTS assay. The results indicated that eudesmin did not exhibit cytotoxicity within the tested concentration range (0.386–3.86 µg/mL). In contrast, epieudesmin showed a reduction in cell viability to 61.0% at the highest concentration tested (10 µM, equivalent to 3.86 µg/mL), as shown in Figure 5.

## 3. Discussion

Previous studies have identified the biological functions of eudesmin, including its cytotoxic, antifungal, and antibacterial effects against Gram-positive and Gram-negative bacteria such as *H. pylori* [26]. However, the effects of eudesmin on infection caused by this microorganism has not been comprehensively investigated [29]. The activity of this natural compound against *H. pylori* is remarkable, but the current literature reports that its action can be affected by epimerization induced by the stomach environment where this bacterium grows [29,30]. However, our results demonstrate that epieudesmin exhibits comparable MIC values but significantly faster bactericidal kinetics against *H. pylori* (Table 2 and Figure 2).

It is currently known that certain virulence genes, such as *cagA* and *vacA* of *H. pylori*, are related to gastric pathologies. Although we were unable to obtain histological diagnoses from the biopsies where the *H. pylori* strains originated, it was observed that only strain ATCC 43504 was *cagA*-positive and *vacAs1/m1*-positive, whereas all clinical strains were negative. Boukhris et al. (2013) reported a significant association between intestinal metaplasia and the *vacAs1/m1* genotype, which is also associated with gastric cancer [31].

Wild *H. pylori* strains (72A, 216A, and 239A) were identified as carrying the *vacA s2/m2/i2* genotype. A significant association was observed between the presence of *cagA* and the *vacA s1/m1/i1* genotype, as well as between the absence of *cagA* and the *vacA s2/m2/i2* genotype, which is consistent with previous reports and like our observations despite the limited number of bacterial strains analyzed [32]. In contrast, both the control strain ATCC 43504, isolated from a patient with gastric cancer, and the multidrug-resistant strain 216A were positive for babA2. Strains harboring babA2 exhibit greater adherence capacity compared to babA2-negative strains. This increased adherence has been associated with elevated levels of lymphocytic infiltration, glandular atrophy, intestinal metaplasia, and enhanced epithelial proliferation [33].

The results obtained by performing the MIC of *H. pylori* with eudesmin and epieudesmin show that bacterial strains are inhibited at different concentrations of these molecules, and that the concentration of eudesmin and epieudesmin required to inhibit *H. pylori* is lower compared to the MICs obtained for antibiotics. These results are relevant because an increase in the failure rate of *H. pylori* eradication therapy, particularly with CLR, has been observed worldwide [7,34], This suggests that conventional triple therapy and alternative therapies may not be effective in different countries [19]. Various susceptibility studies have been conducted to evaluate the effectiveness of treatments in different regions of the world, mainly with CLR, where the therapeutic success rate decreases to 40% and up to 85% if antibiotic resistance is present [35,36]. In Europe, Megraud et al. (2013) reported a primary resistance rate in the adult population of 17.5% [34]. Furthermore, overall CLR resistance rates have reached 30% in Italy and Japan, 40% in Turkey, and 50% in China, although rates of 15% have been reported in Sweden and Taiwan [37]. While in Latin America a high resistance of *H. pylori* to first-line antibiotics has been demonstrated, on average 53% resistance to metronidazole and between 12% and 30% resistance to CLR in some countries of the region, this demonstrates the importance of conducting research to develop new therapeutic alternatives against *H. pylori* [38].

The activity of eudesmin against *H. pylori* is reported to be highly effective. However, our results show that epieudesmin exhibits better inhibitory activity against *H. pylori*, contrary to the findings of Bravo-Arrepol et al. (2023), who indicate that eudesmin activity may be affected by the epimerization balance induced by the stomach environment [30]. However, their study evaluated the epimerization activity of eudesmin against parasitic fungal strains, noting that its epimer loses this activity. Although we determined that the epimerization of eudesmin requires more extreme pH conditions and longer reaction times, both compounds can be considered distinct and stable molecules with potent antibacterial activity.

Using MIC values as a reference, bacterial elimination kinetics were calculated (Figure 3) using an MIC concentration of ½ MIC for each strain. The results revealed that epieudesmin exhibited extremely rapid bactericidal activity, with a complete absence of viable cells after only 2 h of exposure. Eudesmin, on the other hand, showed slightly more gradual, though equally effective, kinetics, achieving complete elimination between 4 and 6 h depending on the strain. According to Pearson’s criterion, which defines lethality as a reduction of 99.9% (3 log10) of the initial inoculum, both molecules proved to be highly effective bactericidal agents [39]. Notably, this effect was irreversible, as extended monitoring up to 72 h confirmed the absence of bacterial regrowth, reinforcing the therapeutic potential of these compounds. These findings are consistent with those reported by Yang et al. (2018) [29], who described eudesmin as a compound with potent activity against *H. pylori*. Our data reinforce the hypothesis that these molecules act through a specifically bactericidal mechanism. While Yang et al. reported a Minimum Bactericidal Concentration (MBC) between 2.5 and 10 mM against multidrug-resistant strains (CLR, MTZ, and AMX), they also highlighted remarkable selectivity, as their activity was reduced against other pathogens such as *S. aureus*, *P. aeruginosa*, and *E. coli* (MBC > 320 mM). This selectivity, combined with the rapid killing kinetics observed in our study, positions eudesmin and epieudesmin as promising candidates for targeted treatment of *H. pylori* infections [29].

It is known that around 80% of the world’s bacteria are capable of forming biofilms, and this has been their main means of survival for billions of years [40,41]. Biofilm-forming bacteria adhere to each other using extracellular polymeric substances (EPS) composed of polysaccharides, proteins, and extracellular DNA (eDNA) and share information through the quorum sensing (QS) system, allowing them to live in an organized manner and protect themselves from conditions that compromise their viability, such as the presence of antibiotics in the environment [42]. The formation of *H. pylori* biofilms reduces the effectiveness of conventional eradication treatments [43]. Most antibiofilm agents are isolated primarily from natural products, many of which are secondary metabolites [44]. Therefore, it is not surprising that plant-derived products have a high capacity for biofilm biomass reduction of bacteria such as *H. pylori*. In this study, the CV assay demonstrated a significant reduction in total biofilm biomass. While this method quantifies both the organic matrix and attached cells, the results suggest that eudesmin or epieudesmin effectively prevent the accumulation of biofilm components during the first 24 h of growth. This finding suggests that these two molecules could be potential candidates for treating antibiotic-resistant *H. pylori* infections, since it has been observed that bacteria forming biofilms increase antibiotic resistance up to 1000-fold. Therefore, developing agents that inhibit biofilm formation is crucial [17].

On the other hand, the ability of these two molecules to inhibit the activity of the urease enzyme was evaluated, since certain plant-derived metabolites, such as those from *Gunnera tinctoria* and *Peumus boldus*, have been shown to inhibit urease activity [45,46]. We observed that neither eudesmin nor epieudesmin affects the urease activity of *H. pylori*, ruling out this enzyme as a direct target of these lignans. However, this does not exclude their antibacterial potential against *H. pylori*, as their activity could be associated with other mechanisms of action relevant to *H. pylori*.

## 4. Materials and Methods

### 4.1. General Information

Preparative chromatography (CC) was performed using Merck silica gel 60 (25–100 μm; Aldrich, Santiago, Chile). Analytical thin-layer chromatography (TLC) was performed using Merck Silica Gel 60F254 sheets (Merck, Darmstadt, Germany), with an elution mixture of n-hexane:ethyl acetate = 9:1 and UV light (254 nm) together with molybdophosphoric acid and heating for visualization. Solvents and fractions were concentrated in a Büchi R100 rotavap (Büchi, Uster, Switzerland). Solvents used in this study were distilled prior to use and dried over appropriate drying agents.

### 4.2. Epimerization of Eudesmin

Eudesmin was isolated from nodes of *Araucaria araucana* (Araucariaceae) collected in January 2019 in Villarica, Chile, 39°24′59″ S, 72°01′07″ W. The knots were identified by Dr. Cristian Paz from the Universidad de La Frontera, and a voucher was kept in the Laboratory of Natural Products and Drug Discovery with the code AA-2019. A quantity of 100 g of milled knots was continuously extracted with boiling hexane, followed by cryocrystallization at 4 °C, according to the procedure described by Bravo-Arrepol et al. (2023) [30]. Epieudesmin was obtained via acid-catalyzed epimerization of eudesmin using hydrochloric acid, followed by purification through column chromatography. Briefly, eudesmin (100 mg, 0.27 mmol) was dissolved in methanol (20 mL) in a 50 mL round-bottom flask and stirred magnetically at 37 °C. Aqueous HCl (0.1 M) was added in portions under different experimental conditions to achieve varying pH values (pH 3, 2, 1, and 0.30), in order to evaluate the epimerization rate. After 24 h, a 5 mL aliquot was withdrawn, extracted with chloroform, and analyzed by GC–MS (Figure 1).

The reaction mixture was then stirred for a total of 72 h, yielding an approximately 1:1 mixture of both epimers. The solution was subsequently extracted three times with ethyl acetate (3 × 50 mL). The combined organic phases were dried over anhydrous MgSO_4_ for 4 h, filtered, and concentrated under reduced pressure to afford a viscous residue. This crude material was purified by column chromatography using hexane:ethyl acetate (9:1 *v*/*v*) as the eluent. Fractions were monitored by thin-layer chromatography (TLC), and those with similar profiles were combined and concentrated under vacuum. Each compound was further crystallized at 4 °C, yielding colorless crystals.

### 4.3. Structural Characterization

The structures of the isolated compounds were elucidated by 1D and 2D NMR spectroscopy, as well as GC–MS analysis. ^1^H and ^13^C NMR spectra were recorded in CDCl_3_ using 5 mm NMR tubes at room temperature on a Bruker Avance III 500 MHz spectrometer (Bruker Biospin GmbH, Rheinstetten, Germany). Two-dimensional experiments, including gs-H,H COSY, edited HSQC, and gs-HMBC, were acquired. All spectra were processed using standard Bruker software (TopSpin 4.x, Bruker, Bremen, Germany).

Eudesmin. ^1^H-NMR (400 MHz, CDCl_3_, ppm): 6.91 (H-2, d, J = 1.7 Hz); 6.88 (H-6, dd, J = 1.7; 8.3 Hz); 6.84 (H-5, d, J = 8.2 Hz); 4.77 (H-7, d, J = 4.3 Hz); 4.26 (1H, H-9a/b, m); 3.91 (1H, H-9a/b, overlap); 3.90 (3H, s); 3.88 (3H, s); 3.12 (H-8, m). ^13^C-NMR (100 MHz, CDCl_3_, ppm): 149.4 (C-3); 148.8 (C-4); 133.7 (C-1); 118.4 (C-6); 111.2 (C-5); 109.4 (C-2); 85.9 (C-7); 71.9 (C-9); 56.1 (C-10/C-11); 56.1 (C-10/C-11); 54.3 (C-8). CGMS: 386.2 g/mol. Spectra are provided in the Supplementary Material Figures S1–S6.

Epieudesmin: ^1^H-NMR (500 MHz, acetone-d6, ppm): 7.00 (2H, m, H-2, H-2′), 6.92 (4H, m, H-5, H-6, H-5′, H-6′), 4.85 (1H, d, *J* = 6.0 Hz, H-7), 4.39 (1H, d, *J* = 7.0 Hz, H-9′), 4.12 (1H, d, *J* = 9.3 Hz, H-7′a/b), 3.81 (1H, m, H-7′a/b), 3.81, 3.81, 3.80, 3.79 (12H, 4 s, MeO-10, MeO-11, MeO-10′, MeO-11′), 3.77 (1H, m, H-9a/b), 3.41 (1H, m, H-8), 3.20 (1H, t, *J* = 8.7 Hz, H-9a/b), 2.88 (1H, m, H-8′). ^13^C-NMR (125 MHz, acetone-d6, ppm): 150.5, 150.2, 149.9, 149.3 (C-3, C-4, C-3′or C-4′), 135.6 (C-1/1′), 132.6 (C-1/1′), 119.1, 118.6 (2 C, C-6, C-6′), 112.7, 112.6 (C-5, C-5′), 110.9, 110.8 (C-2, C-2′), 88.4 (C-8), 82.6 (C-7), 71.6 (C-7′), 70.2 (C-9), 56.2, 56.1, 56.1, 56.1 (4 C, MeO-10, MeO-11, MeO-10′, MeO-11′), 55.6 (C-8′), 50.9 (C-8). C_22_H_26_O_6_. 386.2 g/mol. Spectra in Supplementary Material (Figures S7–S12).

### 4.4. Toxicity Assays in HepG2 Cells

The purity of eudesmin and epieudesmin was confirmed as greater than 98% by GC-MS and NMR analyses prior to bioassays. Cell viability was assessed using the CellTiter 96^®^ AQueous One Solution Cell Proliferation Assay (MTS) (Promega, Madison, WI, USA) to determine the cytotoxic effects of eudesmin and epieudesmin on HepG2 cells. This assay is based on the bioreduction of a tetrazolium compound into a colored, water-soluble formazan product by mitochondrial dehydrogenases in metabolically active cells. The amount of formazan produced is directly proportional to the number of viable cells present in culture. HepG2 cells were purchased in Merck (Santiago, Chile) with code 85011430.

All experiments were performed according to the manufacturer’s instructions. Briefly, HepG2 cells were seeded into 96-well plates at a density of 5 × 10^3^ cells per well in 100 μL of culture medium and incubated at 37 °C. Cells were then treated with increasing concentrations of eudesmin and epieudesmin (0.1–10 μM equivalent to 0.386–3.86 µg/mL). After 24 h of incubation, 20 μL of MTS reagent was added to each well, followed by an additional 4 h incubation at 37 °C. Absorbance was measured at 490 nm using a microplate reader (VICTOR Nivo^TM^; PerkinElmer, Waltham, MA, USA). Cell viability was expressed as a percentage relative to untreated control cells.

### 4.5. Bacterial Strains

The *H. pylori* bacterial strains ATCC 43504 (control), 72A, 216A, and 239A were obtained from the Microbial Collection of the Bacterial Pathogenicity Laboratory at the University of Concepción and stored at −80 °C. Strains 72A, 216A, and 239A were clinical isolates from gastric biopsies of a previous study approved by the Scientific Ethics Committee of the Concepción Health Service, Chile, code CEC 16-05-27. The strains were incubated under microaerobic conditions (10% CO_2_, 5% O_2_, and 85% N_2_) for 5–8 days on Columbia agar (Oxoid, Basingstoke, UK) containing 5% defibrillated horse blood and DENT agar (Oxoid, Basingstoke, UK). In all experiments, *H. pylori* cultures were examined under a microscope (Gram staining) to confirm their rod-shaped form. Additionally, urease, catalase, and oxidase activities were tested to assess the purity and viability of the cultures.

### 4.6. Genetic Characterization of H. pylori

The virulence genotype of *H. pylori* strains was determined by PCR using the primers listed in Table 4. The reaction mix consisted of 25 μL of the final solution, 12.5 μL of SapphireAmp Fast PCR Master Mix (TAKARA BIO INC, Shiga, Japan) 1 μL of each primer at a concentration of 10 μM, 1 μL of DNA template, and 9.5 μL of nuclease-free PCR-grade water. Amplification was performed in an Eppendorf Mastercycler gradient thermocycler using the cycling parameters in Table 4. PCR products were visualized on 1.5% agarose gels, and a 100 bp molecular weight marker was used.

### 4.7. Antibiotic Susceptibility

Antibiotic susceptibility was assessed using the agar dilution method to determine the minimum inhibitory concentration (MIC) of the antibiotics clarithromycin (CLR), metronidazole (MTZ), levofloxacin (LEV), amoxicillin (AMX), and tetracycline (TET), according to the Clinical and Laboratory Standards Institute (CLSI) M100 guidelines [51]. MIC values were determined based on the 9th version of the EUCAST clinical cut-off point (2025) [52]. Resistant strains were defined as >0.25 µg/mL for CLR, >8 µg/mL for MTZ, >1 µg/mL for LEV, >0.125 µg/mL for AMX and >1 µg/mL for TET. *H. pylori* ATCC 43504 was used as a control strain according to CLSI [51].

### 4.8. Antibacterial Activity Against H. pylori

The MIC of eudesmin and epieudesmin was evaluated using the agar dilution method with different concentrations. Two microliters of a McFarland 2 suspension (6 × 10^8^ colony-forming units (CFU)/mL) were plated onto Mueller–Hinton agar plates supplemented with 5% equine blood and incubated at 37 °C under microaerobic conditions. Bacterial clearance kinetics were performed over a 72 h period. Aliquots were taken for viable cell count (CFU/mL) at 0, 2, 4, 8, 16, 32, 48, and 72 h intervals. The MIC was defined as the plate containing the lowest concentration of eudesmin or epieudesmin that showed no visible bacterial growth.

### 4.9. Combined Antibacterial Effect Against H. pylori

The checkerboard dilution method was used to evaluate the effects of eudesmin, epieudesmin, and the antibiotics CLR, MTZ, and LEV against *H. pylori*. Mueller–Hinton broth was used as the culture medium, which was added to a 96-well plate, and serial dilutions were performed to obtain different concentrations of the drug combination in each well. Quantities of 10 μL of bacterial solutions (working concentration of 1 × 10^9^ CFU/mL per well) were added to each well, and the plates were incubated for 72 h at 37 °C under microaerobic conditions. This test was performed in triplicate. The fractional inhibitory concentration (FIC) was calculated as follows: <0.5, synergistic; 0.5–1, additive; 1–2, indifferent; >2, antagonistic; and was calculated using the following formula:

$$FIC=\frac{MIC\;of\;drug\;A\;in\;combination}{MIC\;of\;drug\;A\;alone}+\frac{MIC\;of\;drug\;B\;in\;combination}{MIC\;of\;drug\;B\;alone}$$

### 4.10. Time-Kill Kinetics

For this assay, *H. pylori* strains were independently exposed to specific concentrations of eudesmin or epieudesmin (based on their respective MICs). After reaching the exponential phase in Brain Heart Infusion (BHI) medium supplemented with 1% yeast extract, the initial inoculum was adjusted to a turbidity equivalent to the McFarland 7 standard (2.1 × 10^9^ CFU/mL). Cultures were incubated at 37 °C under microaerobic conditions for 72 h. To determine kill kinetics and assess bacterial regrowth potential, viability counts were performed at 0, 2, 4, 8, 16, 32, 48, and 72 h intervals by microdroplet technique. Briefly, 100 µL aliquots were taken from each culture and serially diluted in phosphate-buffered saline (PBS). Subsequently, 10 µL of each dilution was plated in triplicate onto Columbia agar plates supplemented with 5% equine blood and DENT supplement (OXOID, UK). The plates were incubated for 72 h at 37 °C under microaerobic conditions. The limit of detection (LOD) was established at 10 CFU/mL. This assay was performed independently in triplicate. The number of colony-forming units (CFU) was counted, and the results were expressed as CFU/mL using the following formula [53].

$$CFU/mL=\frac{average\;of\;counted\;CFU\times dilution\;factor}{seeded\;volume\;expressed\;in\;mL}$$

### 4.11. Effect of Molecules on Biofilm Biomass Formation

The crystal violet (CV) staining method was used [54]. CV staining was performed according to the procedure described by Ramos et al. (2012), with minor modifications [55]. These modifications included the use of microtiter plates and the introduction of a drying step following CV staining. After culturing the bacterial strains on Columbia agar plates for 72 h at 37 °C under microaerobic conditions, bacterial suspensions were prepared at concentration of 2 McFarland (6 × 10^8^ CFU/mL) in liquid culture medium (Brucella supplemented with 1% yeast extract). Once the bacterial suspensions were prepared, sterile round glass coverslips were placed in 24-well flat-bottom plates (12 mm diameter per well). A quantity of 700 µL of inoculum of each bacterial suspension was placed on coverslips, and 1/2 of the MIC of eudesmin or epieudesmin was added to each bacterial strain at the start of the assay to evaluate the ability of the compounds to prevent biofilm formation by inhibiting initial bacterial adhesion and early maturation. Subinhibitory concentrations (1/2 MIC) were selected to evaluate the effect of the molecules on biofilm biomass formation, using the following concentrations for eudesmin and epieudesmin, respectively: for strain ATCC 43504, 0.78 µg/mL and 0.39 µg/mL; for strain 72A, 0.78 µg/mL and 1.56 µg/mL; for strain 216A, 0.39 µg/mL and 0.39 µg/mL; finally for strain 239A, 0.78 µg/mL and 0.78 µg/mL, without affecting primary bacterial viability, as determined in the previous MIC assays (Table 2). The plates containing the bacterial suspensions were incubated at 37 °C under microaerobic conditions for 24 h. After incubation, the medium and planktonic cells were removed by aspiration, and the wells were washed three times with PBS. The wells were allowed to air dry at room temperature for 10 min, and 800 µL of 0.1% *w*/*v* CV (Merck, Darmstadt, Germany) were added for 5 min at room temperature. Unbound CV was discarded, and the wells were allowed to air dry for another 10 min at room temperature. Four washes with PBS were then performed to remove excess free CV. The dye bound to the biofilm was extracted by adding 800 µL of an ethanol/acetone mixture (80:20 *v*/*v*), and biofilm formation levels were estimated by measuring the absorbance of the solution at 590 nm using an Infinite M200 Pro microplate reader (Tecan Trading AG, Männedorf, Switzerland). The culture medium containing the bacteria but without the compound is used as a control. The OD was normalized by subtracting the absorbance of the blank control (sterile medium with the molecules) from the absorbance of the inoculated samples to account for any turbidity inherent to the molecules. This assay was performed in triplicate, followed by three technical replicates (9 assays in total).

#### Scanning Electron Microscopy (SEM)

Scanning electron microscopy (SEM) was used to visualize *H. pylori* cells [54]. The same procedure as in the kill kinetics assay described above was followed. *H. pylori* strains were incubated in Brucella-eudesmin or Brucella-epieudesmin broth for 24 h, washed three times with PBS, and fixed with 2.5% glutaraldehyde for 48 h at 4 °C. The samples were then washed with PBS and dehydrated using a graded ethanol series (50%, 70%, 80%, 90%, 100%) at room temperature. Finally, the cells were visualized using an Auto-scan U1 scanning electron microscope (ETEC Corporation, Washougal, WA, USA) at the Microscopy and Spectroscopy Center of the University of Concepción, Chile.

### 4.12. Urease Inhibition Assay

To extract urease, *H. pylori* J99 strain was cultured in the conditions described above. The bacteria were harvested and suspended in sterile distilled water. The suspension was frozen and thawed seven times, using 60 s of sonication in each cycle of thawing. Following centrifugation (8000 rpm for 10 min), the urease of the supernatant was precipitated with ammonium sulfate at 60%. The suspension was centrifuged and the precipitate was resuspended in 4 mL of water and was desalted through a Sephadex G-25 column (Sigma, Darmstadt, Germany). Fractions were collected and those that showed positive urease activity were pooled and mixed with an equal volume of glycerol and stored at −20 °C until use [56].

Evaluation of urease activity was carried out according to a previously published methodology [57]. *H. pylori* and *Canavalia ensiformis* urease (Jack Bean urease, Sigma, Darmstadt, Germany) were used, separately, in the assay mixture (25 μL, 4U) with 25 μL of different concentrations of eudesmin and epieudesmin according to the MIC of each *H. pylori* strain. Samples and urease were preincubated for 4 h at room temperature (for *H. pylori* urease, it was used at 37 °C) in a 96-well assay plate. After preincubation, 200 μL of 100 mM phosphate buffer at pH 6.8 containing 500 mM urea and 0.002% phenol red was added. The measurement was performed by absorbance at 570 nm using the Infinite M200 PRO instrument (TECAN, Männedorf, Switzerland). Urease activity was quantified at 570 nm by the colorimetric change of phenol red. Urea hydrolysis generates ammonia, raising the pH and shifting the indicator towards its deprotonated form (fuchsia), whose maximum absorbance occurs at this wavelength. Thus, absorbance is directly proportional to ammonia production and allows for the determination of the degree of enzyme inhibition of the evaluated molecule. This test was performed in triplicate.

### 4.13. Statistical Analysis

An analysis of variance (ANOVA) with a significance level of *p* < 0.05 and a Bonferroni post hoc test were performed on the data obtained from urease activity and *H. pylori* adhesion, using OD measurements in cultures with *H. pylori* biofilms. In addition, Student’s *t*-test (with a 95% confidence interval) was applied to analyze the differences in *H. pylori* biofilm formation on abiotic surfaces. All analyses were performed using GraphPad Prism 9.0 software (GraphPad Software, La Jolla, CA, USA).

## 5. Conclusions

This study demonstrates that eudesmin is a stable compound in digestive conditions (pH 1 c.a., 37 °C, 4 h), but under strongly acidic conditions, it epimerizes to epieudesmin, and both compounds are capable of inhibiting not only the growth of *H. pylori* but also its biofilm formation. Therefore, it is suggested that eudesmin and epieudesmin could be promising candidates for non-antibiotic adjunctive therapy against *H. pylori*, or their structure could be used for rational design of bioactive analogs against *H. pylori*. Nevertheless, in vivo efficacy studies, pharmacokinetic evaluation, and clinical trials are required to validate the therapeutic potential of these lignans.

## Figures and Tables

**Table 1:** Association of *H. pylori* and its virulence genes.

Virulence Genes	*H. pylori* Strains			
	ATCC 43504	72A	216A	239A
*16S rRNA*	+	+	+	+
*cagA*	+	-	-	-
*dupA*	-	-	-	-
*vacA region m1*	+	-	-	-
*vacA region m2*	-	+	+	+
*vacA region s1a*	+	-	-	-
*vacA region s1b*	-	-	-	-
*vacA region s2*	-	+	+	+
*vacA region i1*	-	-	-	-
*vacA region i2*	-	+	+	+
*babA2*	+	-	+	-

**Table 2:** Minimum inhibitory concentration of eudesmin, epieudesmin and five antibiotics against four *H. pylori* strains.

Compound	MIC (µg/mL) Against *H. pylori* Strains			
	ATCC 43504	72A	216A	239A
Eudesmin	1.56	1.56	0.78	1.56
Epieudesmin	0.78	3.12	0.78	1.56
**Antibiotics**				
Clarithromycin	0.015	0.12	2	2
Metronidazole	64	0.5	64	2
Levofloxacin	0.125	1	4	4
Amoxicillin	0.015	0.015	0.0625	0.0312
Tetracycline	0.125	0.0625	0.0625	0.0625

**Table 3:** Combination of the effect of eudesmin, epieudesmin and antibiotics on the inhibition of *H. pylori*.

Molecule	Strain	Antibiotic	Stand-Alone MIC (µg/mL)		Combination MIC (µg/mL)		FIC	Interaction
			Molecule	Antibiotic	Molecule	Antibiotic		
Eudesmin	43504	CLR	1.56	0.02	0.10	0.02	1.06	Irrelevant
		MTZ	1.56	64.00	0.78	64.0	1.50	Irrelevant
		LEV	1.56	0.13	0.78	0.12	1.49	Irrelevant
	72A	CLR	1.56	0.12	0.39	0.12	1.25	Irrelevant
		MTZ	1.56	0.50	0.78	0.25	1.00	Irrelevant
		LEV	1.56	1.00	0.78	0.50	1.00	Irrelevant
	216A	CLR	0.78	2.00	0.39	2.00	1.50	Irrelevant
		MTZ	0.78	64.00	0.39	32.00	1.00	Irrelevant
		LEV	0.78	4.00	0.20	4.00	1.25	Irrelevant
	239A	CLR	1.56	2.00	0.39	2.00	1.25	Irrelevant
		MTZ	1.56	2.00	0.39	2.00	1.25	Irrelevant
		LEV	1.56	4.00	0.39	4.00	1.25	Irrelevant
Epieudesmin	43504	CLR	0.78	0.02	0.20	0.02	1.25	Irrelevant
		MTZ	0.78	64.00	0.78	32.00	1.50	Irrelevant
		LEV	0.78	0.13	0.78	0.06	1.50	Irrelevant
	72A	CLR	3.12	0.12	1.56	0.06	1.00	Irrelevant
		MTZ	3.12	0.50	0.39	0.50	1.13	Irrelevant
		LEV	3.12	1.00	0.20	1.00	1.06	Irrelevant
	216A	CLR	0.78	2.00	0.39	2.00	1.50	Irrelevant
		MTZ	0.78	64.00	0.39	64.00	1.50	Irrelevant
		LEV	0.78	4.00	0.20	4.00	1.25	Irrelevant
	239A	CLR	1.56	2.00	0.39	2.00	1.25	Irrelevant
		MTZ	1.56	2.00	0.78	1.00	1.00	Irrelevant
		LEV	1.56	4.00	0.78	2.00	1.00	Irrelevant

**Table 4:** Primers used in the detection and genotyping of *H. pylori* from cultures taken from gastric biopsies.

Gen	Region	Sequence	TM (°C)	pb	Reference
*ARNr 16S*		F-5′CTC GAG AGA CTA AGC CCT CC 3′	53	119	[47]
		R-5′ATT ACT GAC GCT GAT GTG C 3′			
*cagA*		F-5′GAT AACAGGCAAGCTTTTGAGG 3′	55	349	[48]
		R-5′CTGCAAAAGATTGTTTGGCAG A3′			
*dupA*		F-5′ACAAGG ACG ATT GAG CGA TGG 3′	61	515	[47]
		R-5′TGG CTA GTT TGA GGT CTT AGG 3′			
*vacA*	*i1*	5′-GTT GGG ATT GGG GGA ATG CCG-3′	55	426	[49]
		5′-TTA ATT TAA CGC TGT TTG AAG-3′			
*vacA*	*I2*	5′-GTTGGGATTGGGGGAATGCCG-3′	55	432	[49]
		5′-GATCAACGCTCTGATTTGA-3′			
*vacA*	*m1*	F-5′ GGTCAAAATGCGGTCATG G 3′	50	290	[50]
		R-5′ CCA TTG GTA CCT GTA GAA AC 3′			
*vacA*	*m2*	F-5′ GGAGCCCCAGGAAACATT G 3′	55	352	[50]
		R-5′ CAT AAC TAG CGCCTTGCA C 3′			
*vacA*	*s1a*	F-5′GTCAGCATCACACCGCAAC 3′	55	190	[50]
		R-5′CTGCTTGAATGCGCCAAAC 3′			
*vacA*	*s1b*	F-5′AGCGCCATACCGCAAGAG 3′	55	187	[50]
		R-5′CTGCTTGAATGCGCCAAAC 3′			
*vacA*	*s2*	F-5′GCTAACACGCCAAATGATCC 3′	55	199	[50]
		R-5′CTGCTTGAATGCGCCAAAC 3′			
*babA2*		F-5′AATCCAAAAAGGAGAAAAAGTATGAAA3′	55	832	[33]
		R-5′TGTTAGTGATTTCGGTGTAGGACA 3′			

## Data Availability

Additional information is provided in the manuscript’s Supplementary Materials.

## Supplementary Materials

The following supporting information can be downloaded at: https://www.mdpi.com/article/10.3390/ijms27146295/s1.
