# Supplementary material for: Acid-Induced Epimerization of Eudesmin to Epieudesmin: Rapid Bactericidal and Antibiofilm Activity Against Helicobacter pylori
Source: Int J Mol Sci. 2026 Jul 15;27(14):6295. doi: 10.3390/ijms27146295 (PMC13411828; doi:10.3390/ijms27146295)
Supplement: Supplementary file 1 [file ijms-27-06295-s001.zip › ijms-4369271-supplementary.pdf]

International Journal of Molecular Sciences

MDPI

Supplementary Material

Acid-Induced Epimerization of Eudesmin to Epieudesmin: Rapid Bactericidal and Antibiofilm Activity against *Helicobacter pylori*

Cristian Parra-Sepúlveda [1-2], Kimberly Sánchez-Alonzo [1], José Becerra [3], Leandro Ortiz [4], Viviana Burgos [5], Cecilia Villegas[6], Felipe A. Sanhueza[7], Benjamín Oporto[7], Cristobal Goic[1], Apolinaria García-Cancino [1*], and Cristian Paz [7*]

[1] Laboratory of Bacterial Pathogenicity, Faculty of Biological Sciences, University of Concepcion, Concepcion Bio Bio 4030000, Chile; cparras@udec.cl (C.P.S); kimsanchez@udec.cl (K.S.A.); cmanzor2018@udec.cl (C.G.)

[2] Doctorado en Ciencias mención Biología Celular y Molecular Aplicada, Universidad de la Frontera, Chile

[3] Universidad de Concepción, Departamento de Botánica, Facultad de Ciencias Naturales y Oceanográficas, Concepción, Chile; jbecerra@udec.cl (J.B)

[4] Instituto de Ciencias Químicas, Facultad de Ciencias, Universidad Austral de Chile, Casilla 567, Valdivia, Chile. leandro.ortiz@uach.cl (L.O.)

[5] Escuela de Tecnología Médica, Centro de Investigación en Prevención y Cuidados de la Salud (+SALUD), Facultad de Salud, Universidad Santo Tomás, Temuco 4780000, Chile; vburgos7@santotomas.cl (V.B)

[6] Departamento de Ciencias Químicas y Biológicas, Facultad de Recursos Naturales, Universidad Católica de Temuco, Rudecindo Ortega, Temuco, Chile; cecilia.villegas@uct.cl (C.V)

[7] Laboratory of Natural Products & Drug Discovery, Department of Basic Sciences, Faculty of Medicine, Universidad de La Frontera, Temuco 4811230, Chile. felipesanhueza2017@gmail.com (F.A.S); b.oporto02@ufromail.cl (B.O)

* Correspondence: C.P, cristian.paz@ufrontera.cl. Tel: +56 45 259 2825; A.G.C. apgarcia@udec.cl. Tel: +56 41 266 4144

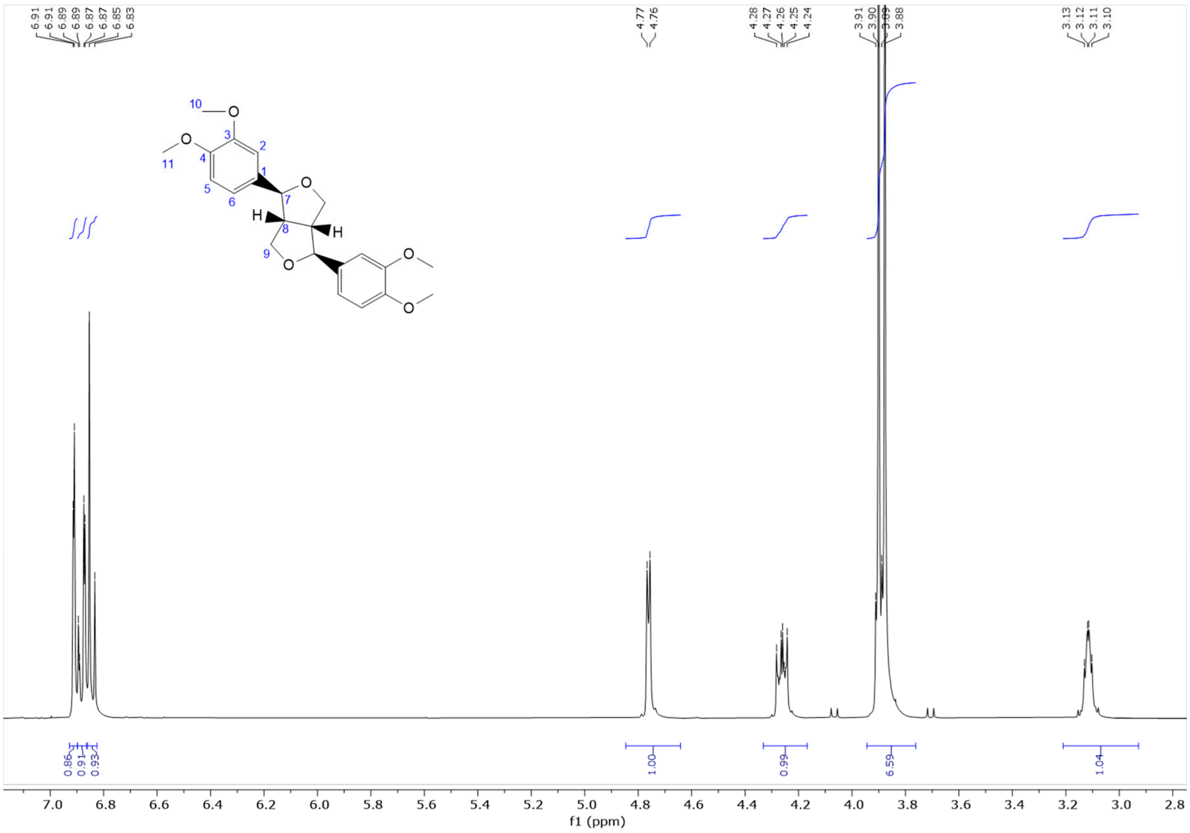

Figure S1. ^{1}H NMR (400 MHz, $CDCl_3$, ppm) of eudesmin. Signal assignments are given in the Materials and Mehods (4.3. Structural Characterization).

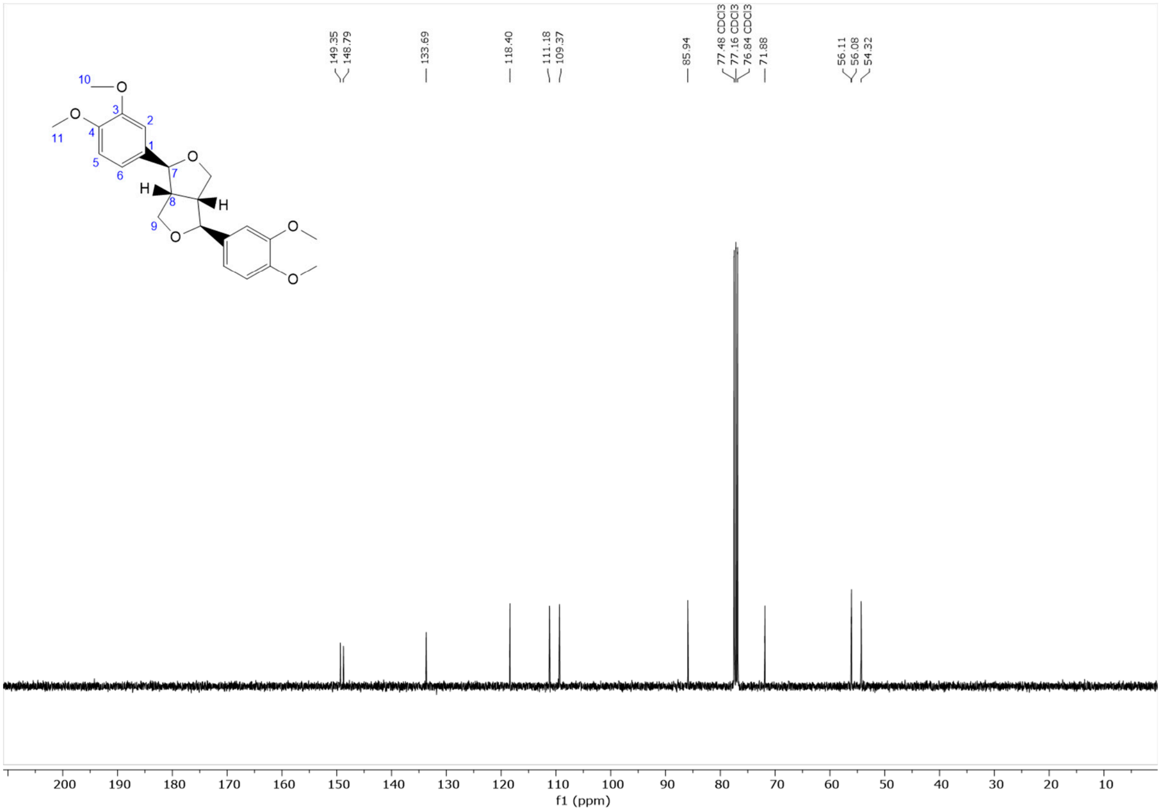

Figure S2. ^{13}C NMR (100 MHz, $CDCl_3$, ppm) of eudesmin. Signal assignments are given in the Materials and Mehods (4.3. Structural Characterization).

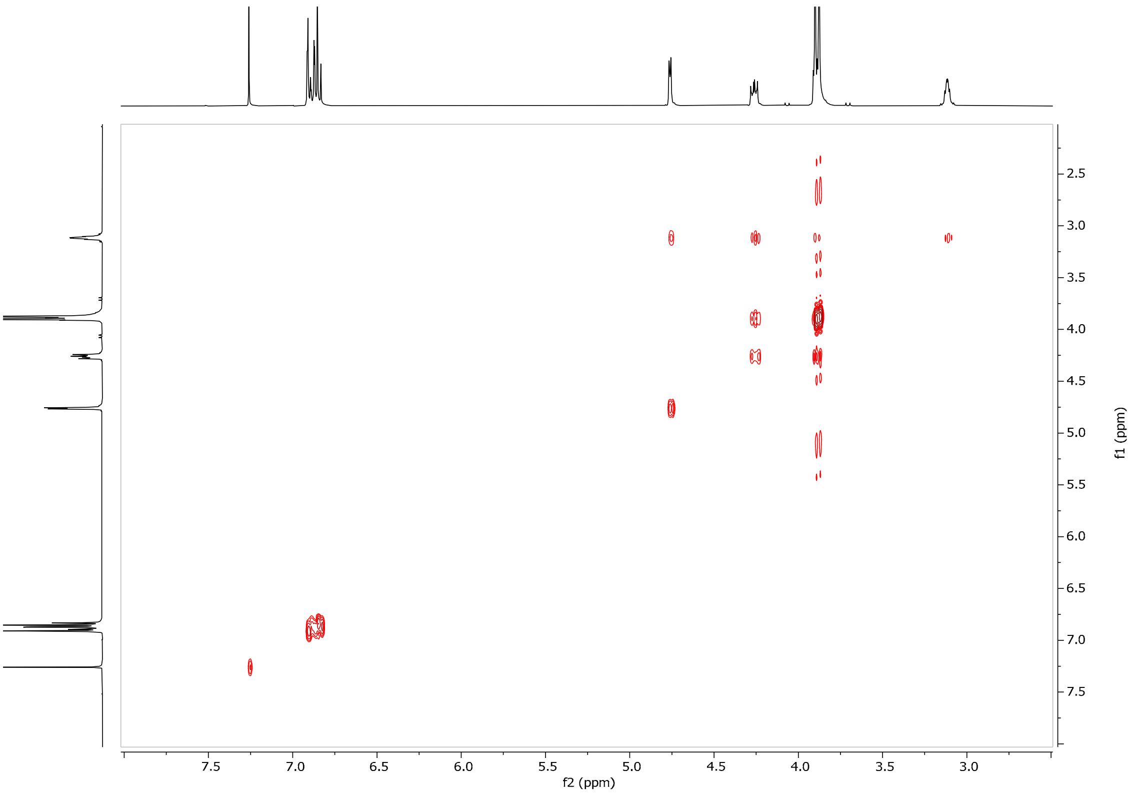

Figure S3. ^{1}H, ^{1}H COSY NMR spectra of eudesmin

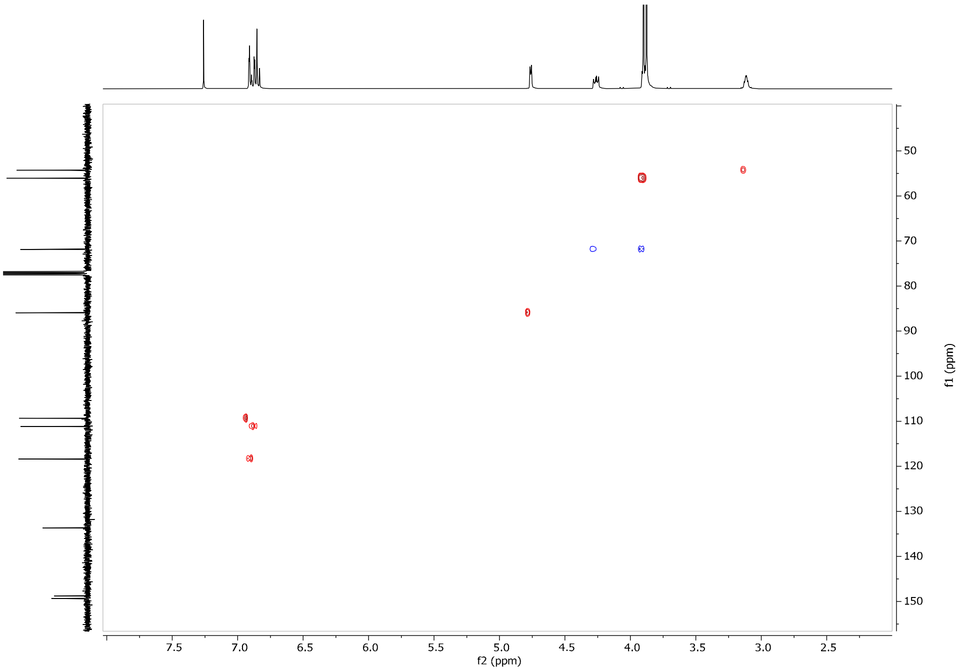

Figure S4. HSQC NMR spectra of eudesmin

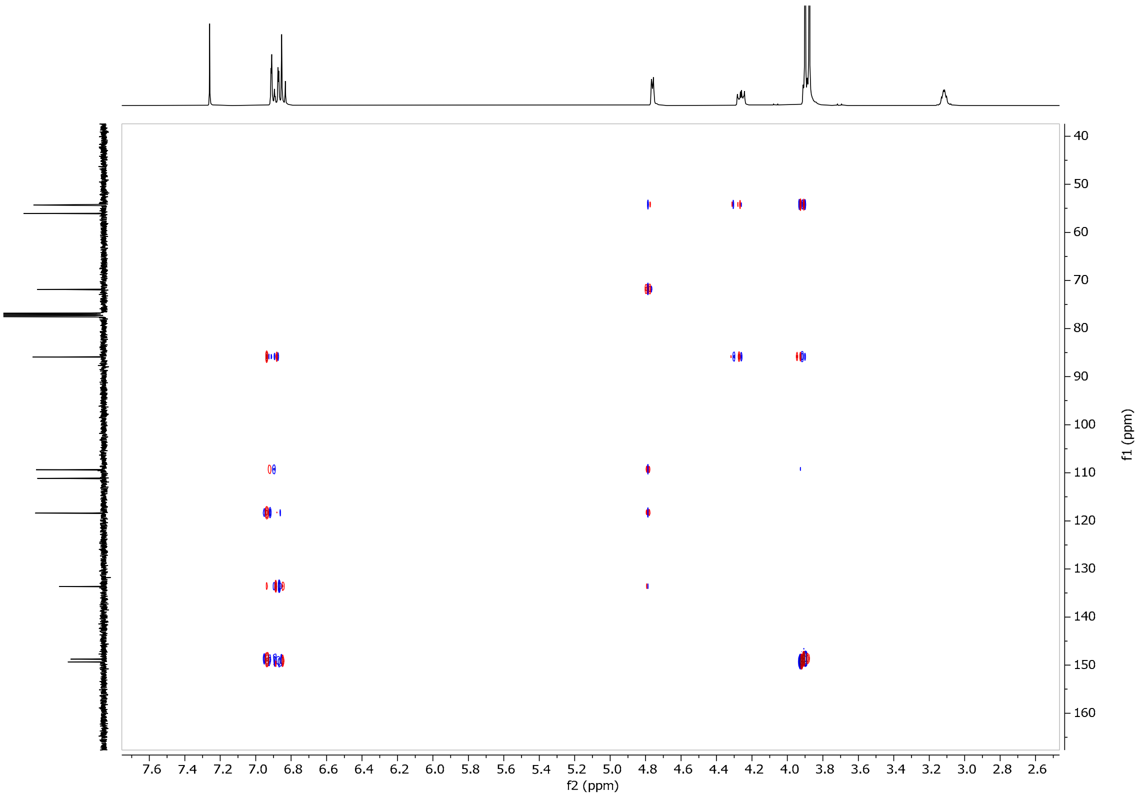

Figure S5. HMBC NMR spectra of eudesmin

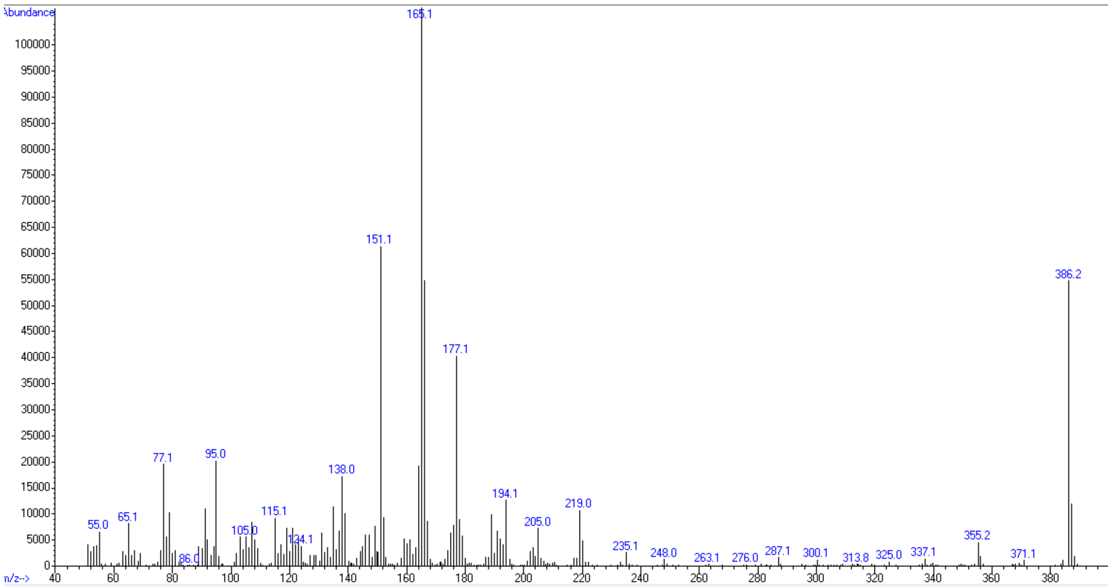

Figure S6. GC-MS spectra of eudesmin

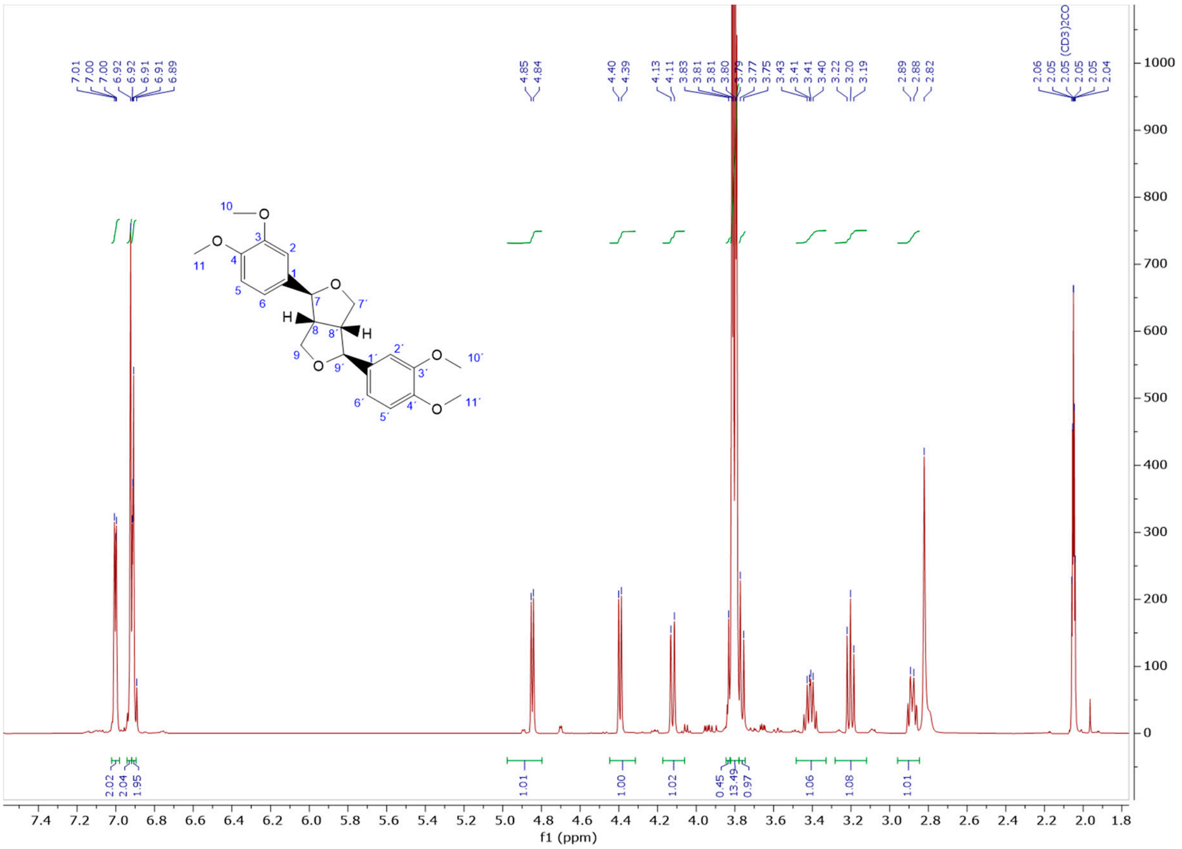

Figure S7. ^{1}H NMR (500 MHz, acetone-d6, ppm) of epieudesmin. Signal assignments are given in the Materials and Mehods (4.3. Structural Characterization).

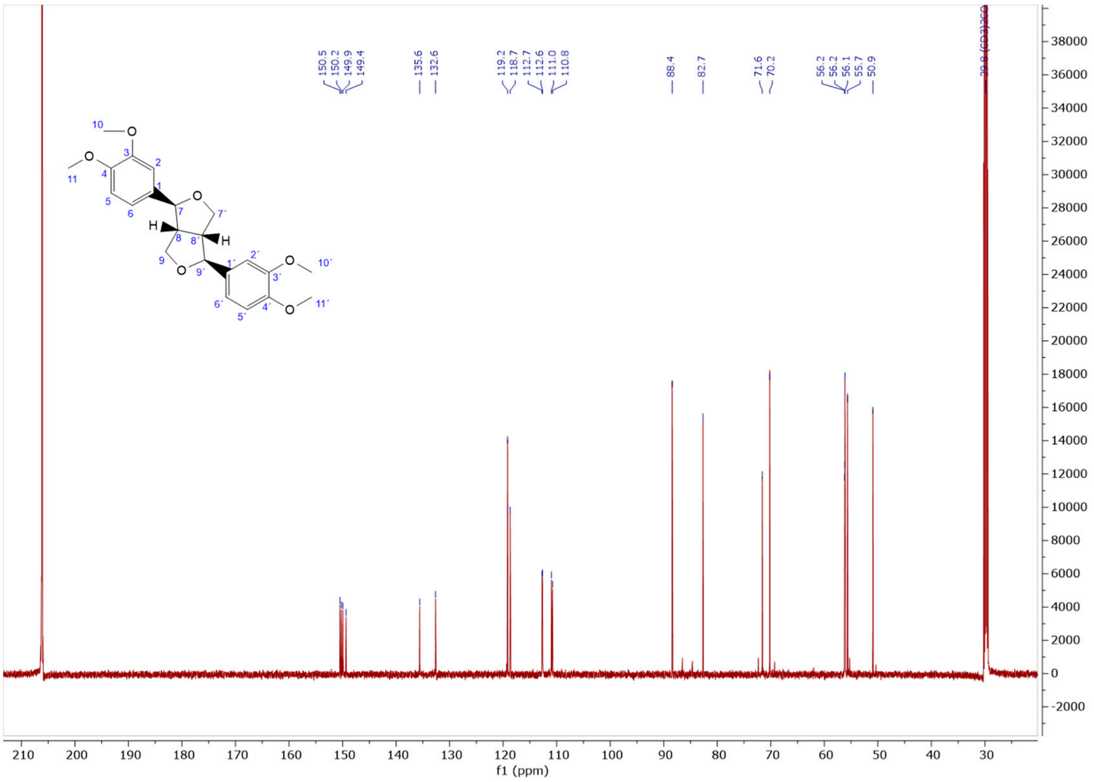

Figure S8. ^{13}C NMR (125 MHz, acetone-d6, ppm) of epieudesmin. Signal assignments are given in the Materials and Mehods (4.3. Structural Characterization).

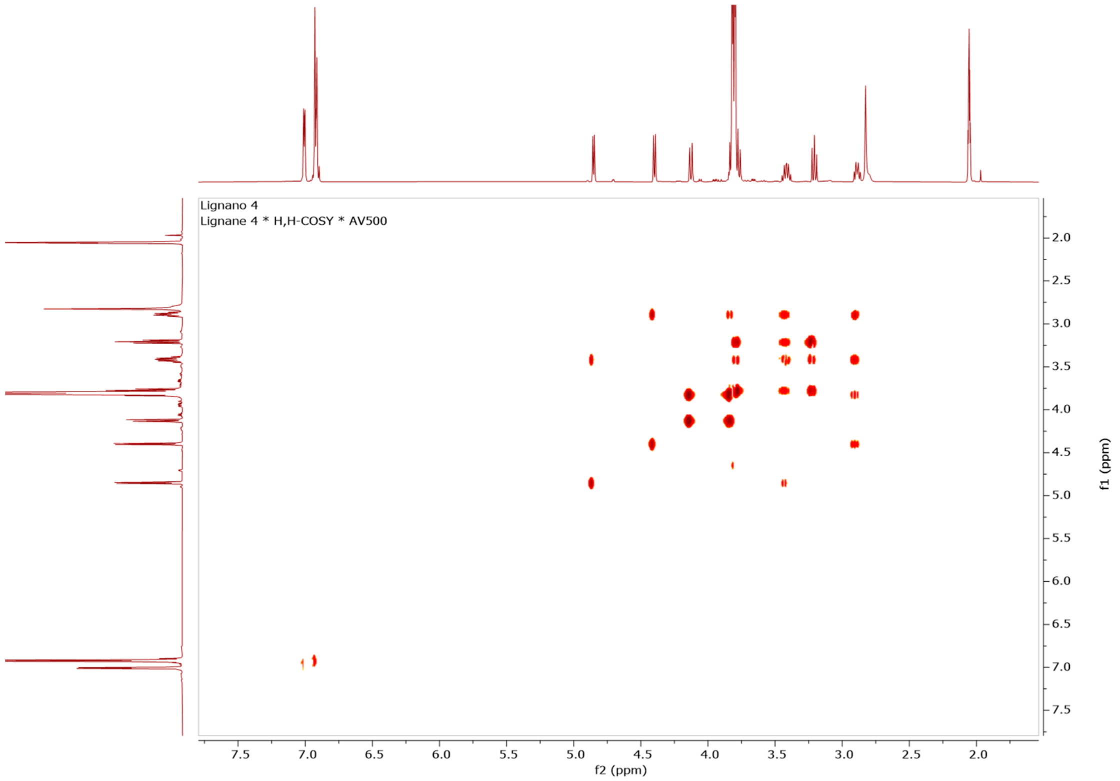

Figure S9. ^{1}H, ^{1}H COSY NMR spectra of epieudesmin

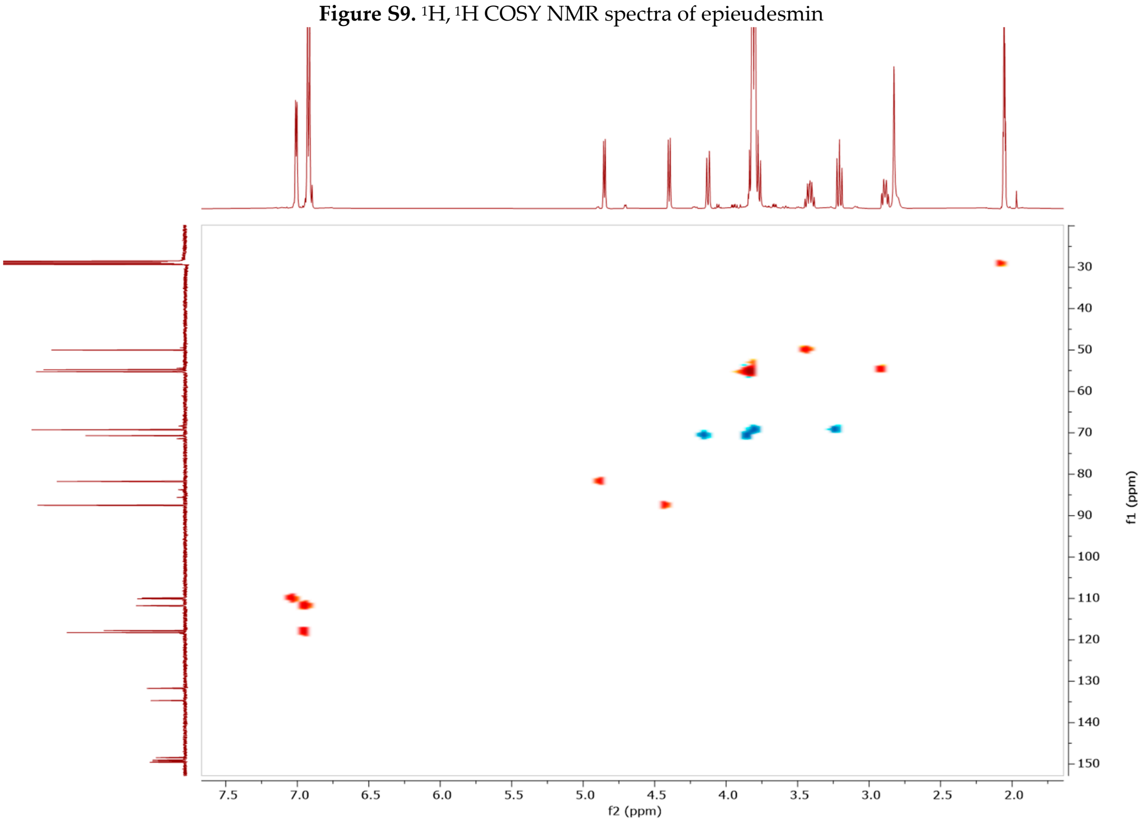

Figure S10. HSQC NMR spectra of epieudesmin

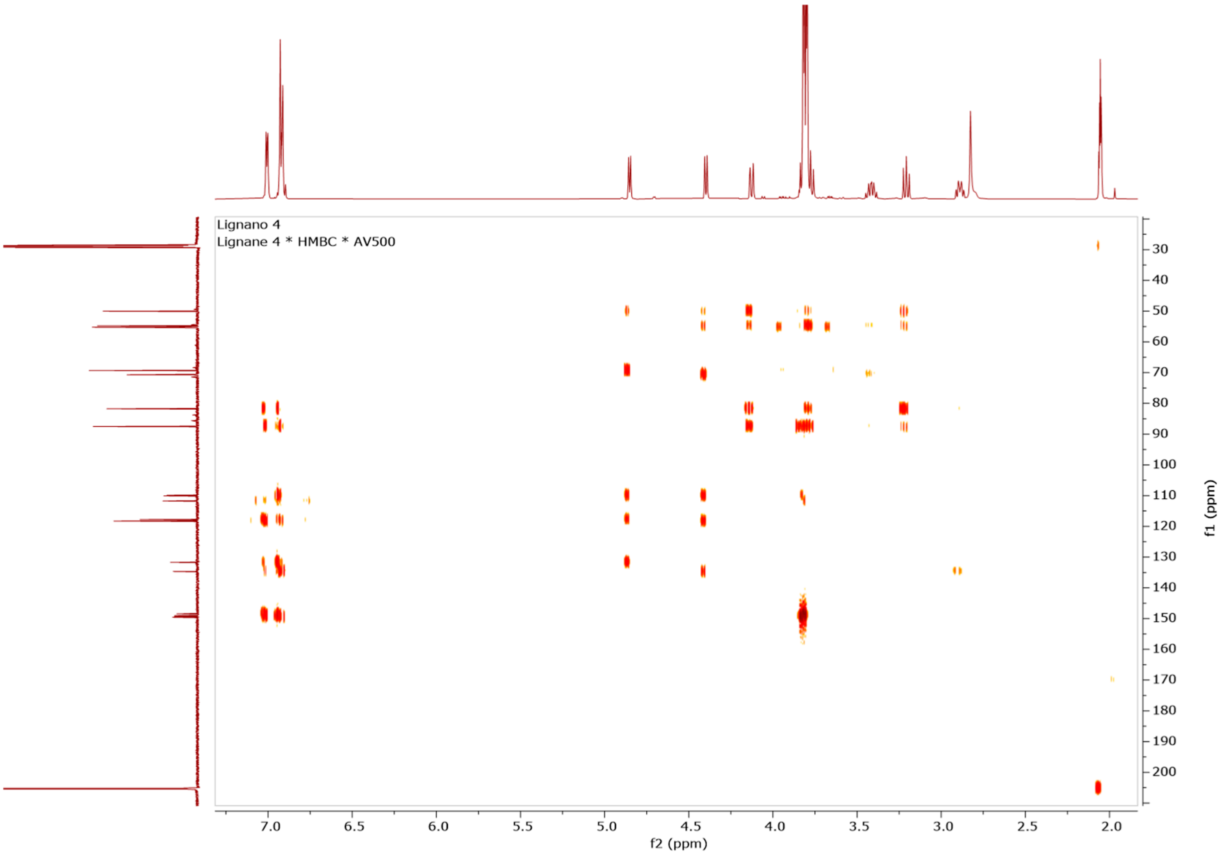

Figure S11. HMBC NMR spectra of epieudesmin

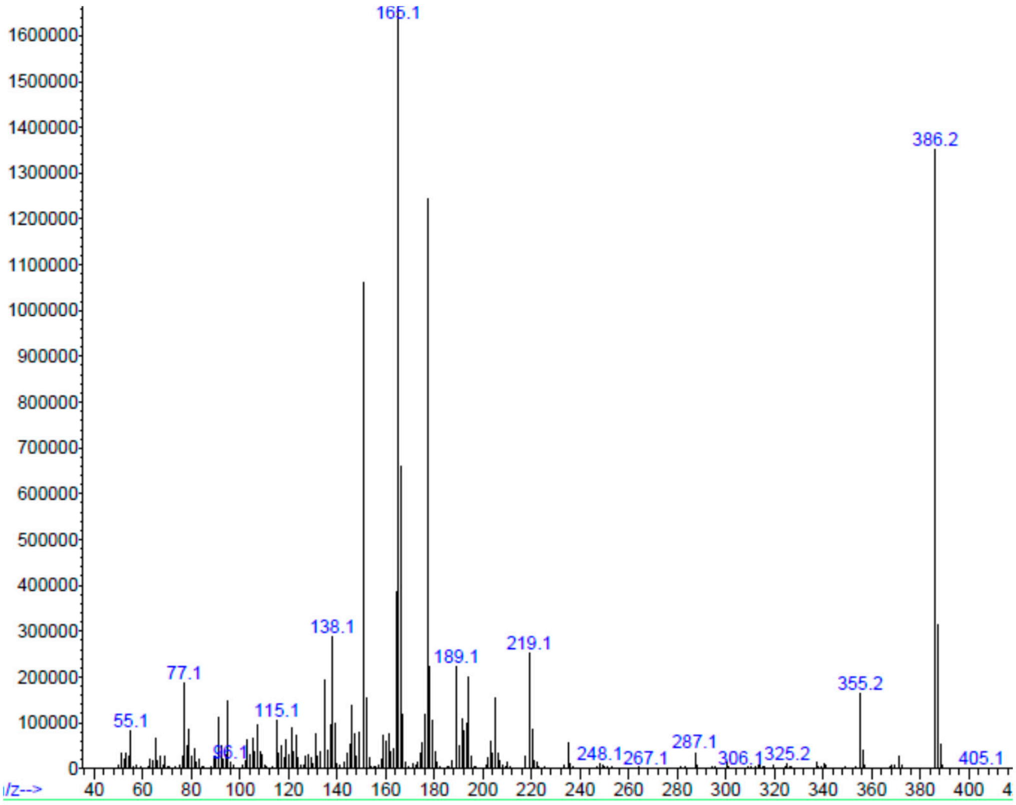

Figure S12. GC-MS spectra of epieudesmin
